# Identification and analysis of short indels inducing exon extension/shrinkage events

**DOI:** 10.1002/2211-5463.13871

**Published:** 2024-07-31

**Authors:** Zhuo Qu, Narumi Sakaguchi, Chie Kikutake, Mikita Suyama

**Affiliations:** ^1^ Division of Bioinformatics, Medical Institute of Bioregulation Kyushu University Fukuoka Japan

**Keywords:** exon extension/shrinkage, individual‐specific genome, RNA‐seq, short indels

## Abstract

The search for genetic variants that act as causative factors in human diseases by disrupting the normal splicing process has primarily focused on single nucleotide variants (SNVs). It is worth noting that insertions or deletions (indels) have also been sporadically reported as causative disease variants through their potential impact on the splicing process. In this study, to perform identification of indels inducing exon extension/shrinkage events, we used individual‐specific genomes and RNA sequencing (RNA‐seq) data pertaining to the corresponding individuals and identified 12 exon extension/shrinkage events that were potentially induced by indels that disrupted authentic splice sites or created novel splice sites in 235 normal individuals. By evaluating the impact of these abnormal splicing events on the resulting transcripts, we found that five events led to the generation of premature termination codons (PTCs), including those occurring within genes associated with genetic disorders. Our analysis revealed that the potential functions of indels have been underexamined, and it is worth considering the possibility that indels may affect splice site usage, using RNA‐seq data to discover novel potentially disease‐associated mutations.

## Graphical abstract

We used individual‐specific genomes and RNA‐seq data of the 235 corresponding individuals and identified 12 exon extension/shrinkage events that were potentially induced by indels. Among the 12 events, five led to the generation of premature termination codons. We consider that exploring the impact of indels on splice site usage may reveal new findings of potential variant functions.

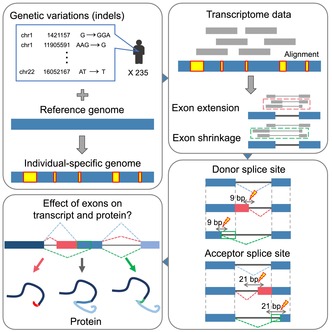

**Abbreviations**
*CMT2A*
: Charcot–Marie–Tooth type 2A
*DECR1*
: 2,4‐dienoyl‐CoA reductase 1
GTF: General Transfer Format
IGV: Integrative Genomics Viewer
Indels: insertions or deletions
*MFN2*
: mitofusin 2
NMD: nonsense‐mediated decay
PTCs: premature termination codons
RNA‐seq: RNA sequencing
SNVs: single nucleotide variants
*TRAPPC2L*
: trafficking protein particle complex subunit 2L
VCF: Variation Call Format

The RNA splicing process plays a crucial role in the regulation of gene expression by generating various mRNA and protein isoforms via diverse splicing patterns. Variants that can affect normal splicing events have been reported not only to be the potential cause of various diseases, including neurodegenerative disorders, cancer, and other genetic disorders [1, 2, 3, 4], but also to be the cause of the generation of new functional protein isoforms related to phenotype [5, 6]. These variants that alter normal splicing are not only located in authentic splice site but may also create novel splice sites by generating splice site sequence motifs. Increasingly, studies have detected single nucleotide variants (SNVs) that affect splice site usage in diseased and healthy samples, some of which can lead to the generation of abnormal transcripts by creating novel splice sites [7, 8, 9, 10, 11]. It is worth noting that insertions or deletions (indels) account for approximately 21% of the total variants within the human genome [12] and have been reported as causative variants through their potential impact on the splicing process [13]. However, there are few such reports, and no comprehensive analysis of the impact of indels on splice site usage has been performed to date.

In this study, as a continuation of our previous study on SNVs‐induced exon extension/shrinkage events [11], to identify the indels that can impact splice site usage, we constructed individual‐specific genomes and transcriptome data reflecting the information of indels and used RNA‐seq data from the corresponding individuals. We identified indels that affected the splicing process by disrupting authentic splice sites or creating novel splice sites. We also evaluated the impact of the abnormal splicing events induced by indels on the resulting transcripts and found that several events resulted in the generation of premature termination codons (PTCs). Our findings suggest that the potential functions of indels have been underexamined, and it is worth considering the possibility that the new findings of potential variant functions can be realized by further exploring the effects of indels on splice site usage.

## Materials and methods

### Construction of an individual‐specific genome

We used individual genomic variation data, which contains both SNVs and short indels, in the Variation Call Format (VCF) from the 1000 Genomes Project [14]. We only used VCF files of the 462 individuals with RNA‐seq data (see the following section “Quality control of RNA‐seq data”). We used reference genome sequence data for the hg19 version from the UCSC Genome Browser [15]. We extracted non‐overlapping indels (the length ranges from 1 to 661 bp) from the individual genomic variation data and constructed an individual‐specific genome that reflected the information on indels for each individual using the original script written in python (version 3.8.17; Python software foundation, Beaverton, OR, USA). In cases in which multiple indels were overlapped, we arranged these indels in ascending positional order and retained the first indel.

### Quality control of RNA‐seq data

We used RNA‐seq data from the lymphoblastoid cell lines of 462 individuals from the GEUVADIS Project [16], which were sampled from the 1000 Genomes Project. As in previous studies [10, 11], we performed a quality check using the fastqc program (https://www.bioinformatics.babraham.ac.uk/projects/fastqc), and only high‐quality RNA‐seq data with sequencing quality scores > 30 were selected by using the original script written in python (version 3.8.17; Python software foundation, Beaverton, OR, USA). We retained the RNA‐seq data from 235 individuals with high‐quality scores for further mapping and identification of novel splicing events.

### Alignment of RNA‐seq data

We used hisat2 (version 2.1.0) [17] to map the RNA‐seq data of each individual to the individual‐specific genome (see “Construction of an individual‐specific genome”) of the corresponding individual. The program's default parameters were used for mapping.

### Construction of individual‐specific transcriptome data

We used reference transcriptome data that documented the gene structure in General Transfer Format (GTF) from the UCSC Genome Browser [15]. g2gtools (version 0.1.31) (https://github.com/churchill‐lab/g2gtools) was used to construct the chain files, which were required for converting the reference transcriptome data into individual‐specific transcriptome data and liftOver [18] was used to construct individual‐specific transcriptome data, comprising the positions of authentic splice sites affected by indels of different lengths for each individual.

### Pipeline to identify indels that induce exon extension/shrinkage events

**Fig. 1:**
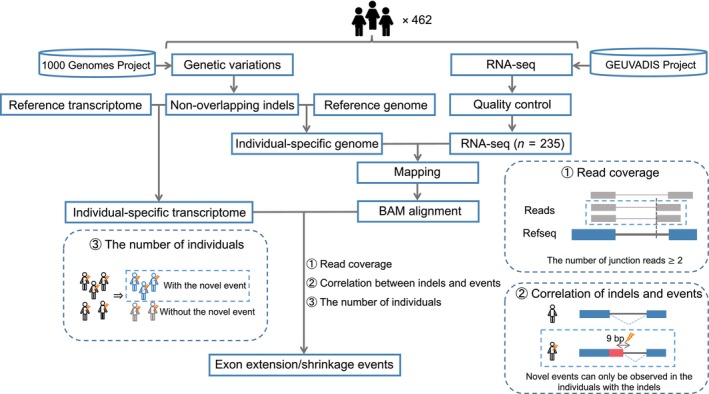
Workflow used for the identification of exon extension/shrinkage events and associated indels. Briefly, genomic variation data from each individual were used to construct an individual‐specific genome and transcriptome, and the RNA‐seq data from the corresponding individual were aligned to the individual‐specific genome. To ensure the accuracy of the novel splicing events identified here and their associated indels, the events were filtered based on three criteria: (1) read coverage, (2) correlation between indels and events, and (3) the number of individuals. BAM, Binary Alignment Map; bp, base pair; indels, insertions or deletions; RNA‐seq, RNA sequencing.

The pipeline we built to identify exon extension/shrinkage events induced by indels is as follows (Fig. 1). We used the aligned junction reads to identify the occurrences of exon extension/shrinkage events. We collected junction reads in which the mapping positions on one side were consistent with the positions of the annotated exons, and in which the mapping positions on the other side were inconsistent with the positions of the annotated exons. Because of the effect of indel occurrences on the shift in genomic coordinates, resulting in distinct positions of the annotated exons among different individuals, we used the positions of authentic splice sites affected by indels from individual‐specific transcriptome data (see “Construction of individual‐specific transcriptome data”). To identify the associated indels that caused the exon extension/shrinkage events, we considered a window of −3/+6 bases at the donor splice site of the novel exon‐intron boundary, as well as −18/+3 bases at the acceptor splice site of the novel boundary. We identified indels located within these regions as candidate indels that were associated with the extension/shrinkage events, regardless of the distance from the authentic original exon‐intron boundary. To ensure the accuracy of the novel splicing events and associated indels identified here, we performed filtering based on the following three criteria. First, we filtered the identified novel splice sites based on read coverage. To reduce mapping errors, we considered sites that were covered by two or more junction reads as candidate novel splice sites. We also applied the following condition: within these junction reads, the side for which the mapping position was inconsistent with the annotated exons must comprise at least 5 bp, thereby further reducing the potential for the occurrence of errors during the mapping process. Next, to ensure that the events are caused by the identified indels, it was essential to confirm that the corresponding novel splicing events were not observed in individuals without the associated candidate indels, because, in such cases, the corresponding novel splicing events are definitely not caused by the candidate indels. Finally, for individuals with candidate indels, if the number of individuals with the corresponding novel splicing events was greater than the number of individuals without them, we retained these candidate indels together with the corresponding novel splicing events. We set this criterion because novel splicing events might not be observed in some individuals, even if they have candidate indels, if the level of expression of the transcript is low.

### Data visualization

We used the Integrative Genomics Viewer (igv) software (version 2.9.4) [19] to visualize the mapping, individual variant, and gene structure data.

### Analysis of the effects of the identified indels on splice sites and transcripts

We used spliceai (version 1.3) [20] to assess whether the identified indels can create novel splice sites. The assessment score ranged from 0 to 1. We checked whether the associated indels were registered in the ClinVar [21] database, to determine if they had already been classified as being pathogenic in previous studies. We explored the potential association between the identified indels and disease pathogenesis by assessing whether the indels were located in genes known to cause genetic diseases. For this purpose, we obtained the list of genes responsible for genetic diseases from the OMIM database [22]. We developed our original scripts using the r software (version 4.2.0; R Foundation for Statistical Computing, Vienna, Austria) to identify exon extension/shrinkage events that can generate PTCs.

### Statistical analyses

We used the r software (version 4.2.0; R Foundation for Statistical Computing, Vienna, Austria) for statistical analyses. The *t*‐test was used to assess whether the SpliceAI scores were significantly different between the identified indels and 10 000 randomly selected indels.

## Results

### Identification and characteristic assessment of exon extension/shrinkage events and their associated indels

**Fig. 2:**
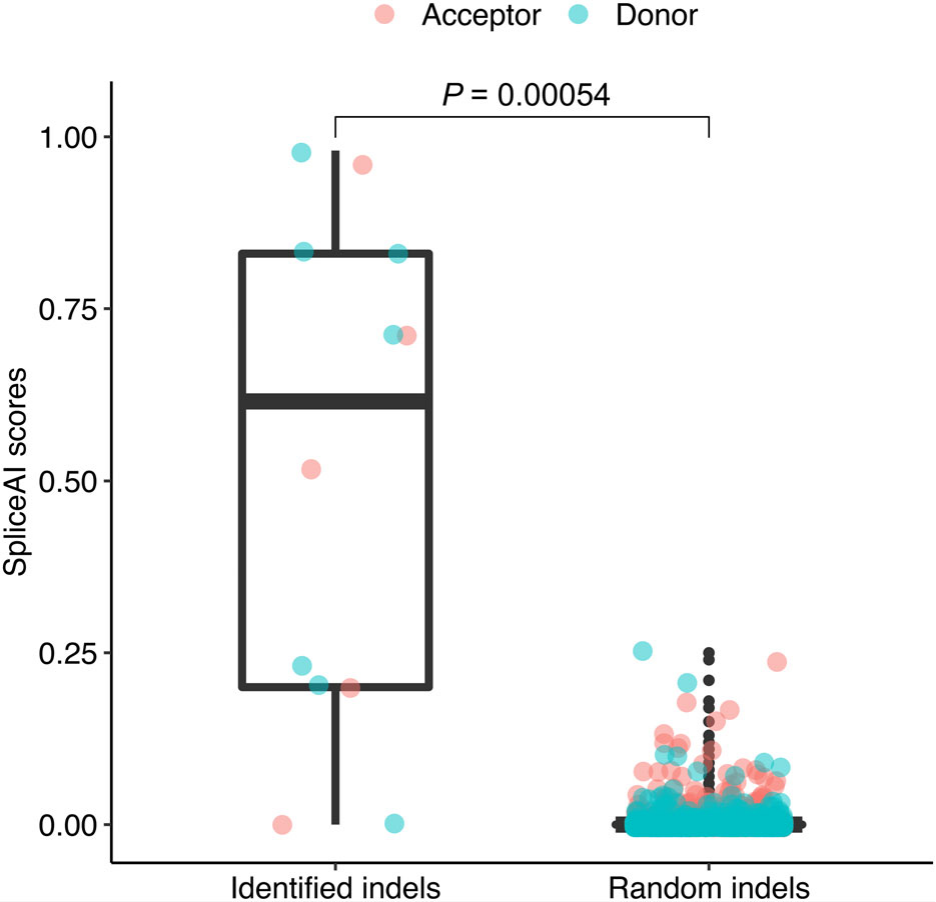
Boxplot of SpliceAI scores for the indels identified here and 10 000 random indels. The indels were categorized into “Acceptor gain” and “Donor gain” (which are depicted in pink and blue, respectively) based on the positions of the splice site created by them. The *t*‐test was used to establish statistical differences. The bottom and top of the box are the 25th and 75th percentiles, the line inside the box is the 50th percentile (median), the upper and lower whiskers represent the maximum and the minimum values of the data. Indels, insertions or deletions.

Applying the pipeline to identify indels that induce exon extension/shrinkage events to genomic variation data and RNA‐seq data of the 235 individuals, we identified 12 exon extension/shrinkage events and their associated indels (seven events were located at donor splice site regions and five were located at acceptor splice site regions), including five extension events and seven shrinkage events (Table 1). Among these, eight indels are located within the authentic splice site regions, while the distance between the identified indels and the authentic splice sites extended up to 62 bp. We found that the SpliceAI scores for most of the indels identified here were significantly higher than those obtained for random indels (*P* = 0.00054) (Fig. 2). Based on the assessment of the impact of the identified extension/shrinkage events on transcripts, we found that, among the 12 novel splicing events identified, five occurred within coding regions. Moreover, all five events had the potential to induce PTC by affecting the reading frame during the translation process. Among these five events, four PTCs were generated within the last exon, considered to be able to potentially escape nonsense‐mediated decay (NMD) process [23], while the remaining one PTC may induce NMD, and we observed that the expression of abnormal transcripts capable of generating the PTC was lower than that of normal transcripts. This could be due to NMD, or stronger expression from authentic splice site, or both. Furthermore, we found that three of the events that induced PTC generation were located within genes that are known to be responsible for genetic disorders listed in OMIM, although only one of these three events had an allele frequency smaller than 0.01. Among the 12 identified associated indels, nine were not reported in ClinVar, whereas the remaining three were classified as being “Benign.”

**Table 1:** List of 12 identified short indels inducing exon extension/shrinkage events.

Position	ID		Ref	Alt	Gene^a^	Splice site^b^	Splicing event^c^	SpliceAI (gain)	PTC	ClinVar	AF^d^	# Samples^e^	
	rs	HGVS										Hetero	Homo
chr1:9,304,978	rs139855605	ENST00000377403.2: c.1‐15_1‐8del	CCCCAGGCA	C	H6PD*	A	S	0.96	Not in CDS	–	0.1199	45	7
chr2:135,626,611	rs201426305	ENST00000392929.2: r.109‐1del	GC	G	*CCNT2‐AS1*	A	S	0.20	Not in CDS	–	0.0139	3	0
chr5:64,103,424	rs551560409	ENST00000508024.1: c.939‐2_939‐1insGC	A	AGC	CWC27*	A	E	0.52	PTC	–	0.0072	2	0
chr8:42,952,662	rs149140769	ENST00000331373.5: c.1‐6029_1‐6026del	TAAAG	T	POMK*	A	S	0.71	Not in CDS	Benign	0.0152	4	0
chr8:91,057,230	rs147668665	ENST00000220764.2: c.885+8del	GT	G	*DECR1*	D	E	0.00	PTC	Benign	0.0131	6	0
chr12:7,086,294	ss1388028816	ENST00000261407.4: c.1461+16del	AC	A	*LPCAT3*	D	S	0.98	Not in CDS	–	0.0182	13	0
chr13:45,957,308	rs35686266	ENST00000517509.1: r.927del	AG	A	*TPT1‐AS1*	D	S	0.23	Not in CDS	–	0.0791	34	2
chr14:101,540,816	rs11310304	ENST00000448840.2: r.354+1del	AC	A	*ENSG00000230805*	D	S	0.20	Not in CDS	–	0.0223	11	0
chr15:65,108,135	rs536092356	ENST00000333425.6: c.1878del	TC	T	*PIF1*	A	S	0.00	PTC	–	0.0101	4	0
chr16:1,822,797	rs4027362	ENST00000397375.2: c.321+2_321+3insACCT	C	CACCT	MRPS34*	D	E	0.83	PTC	Benign	0.4925	119	54
chr16:88,926,388	rs543819824	ENST00000301021.3: c.374+8_374+9insTACTTTCTGTGTCTGCATCCAGTCCAGGTGGGCCT	C	CTACTTTCTGTGTCTGCATCCAGTCCAGGTGGGCCT	TRAPPC2L*	D	E	0.71	PTC	–	0.0565	13	5
chr17:15,343,524	rs71150251	ENST00000522212.2: c.492+45_492+46insCTT	C	CCTT	*TVP23C‐CDRT4*	D	E	0.83	Not in CDS	–	0.9725	6	229

^a^ The genes known to cause genetic disorders listed in the OMIM database are indicated with asterisks

^b^ A, acceptor splice site; D, donor splice site

^c^ E, exon extension; S, exon shrinkage

^d^ AF, allele frequency

^e^ Number of samples with the genotype in the 235 samples analyzed.

### Examples of exon extension/shrinkage events and associated indels

**Fig. 3:**
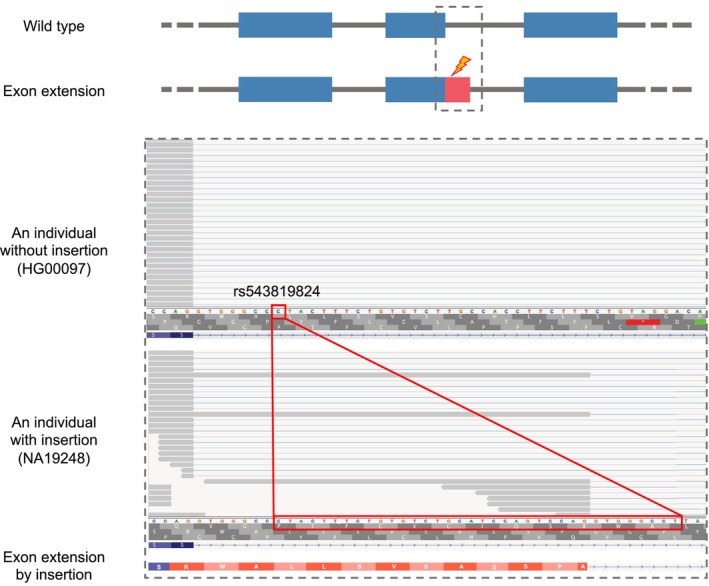
Example of an exon extension event that was identified at the donor splice site of exon 4 (the 4th of five exons) of the trafficking protein particle complex subunit 2L (*TRAPPC2L*) gene, which was induced by an insertion. A schematic diagram of exon extension is shown at the top. The blue segments indicate authentic exons, pink segments indicate extended exons and gray lines indicate introns. The bottom part illustrates the alignment of junction reads around the extended exon in samples with and without the identified indel. The gray segments represent the reads aligned to the genome. The amino acid sequences of the gene before and after the exon extension event are shown below the read alignment. The red box indicates the position of the insertion.

As an example, we selected an exon extension event that was identified at the donor splice site of exon 4 (the 4th of five exons) of the Trafficking Protein Particle Complex Subunit 2L (*TRAPPC2L*) gene (Fig. 3). The identified insertion consisted in a change from C to CTACTTTCTGTGTCTGCATCCAGTCCAGGTGGGCCT, which created a novel donor splice site. Interestingly, 21 bases on the 3′ side of this inserted sequence were duplicates of the authentic splice site, thereby forming a novel downstream splice site. The SpliceAI score of this insertion was 0.71, indicating a higher probability of creating a novel splice site compared with other random indels (Fig. 2). The extended exon was 35 bp longer than the annotated exon, and the extension event induced a frameshift, thereby introducing a PTC at codon 145. As in this example, when the activated novel splice site was located within the nucleotides that were directly affected by the indels, it was difficult to identify these abnormal splicing events by mapping the RNA‐seq data to the reference genome as it is (Fig. S1), showing the importance of mapping against individual‐specific genomes.

**Fig. 4:**
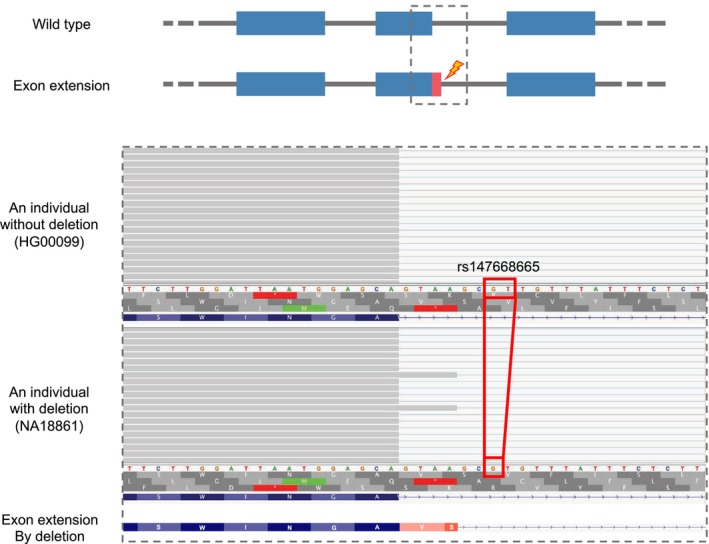
Example of an exon extension event identified at the donor splice site of exon 8 (the 8th of 10 exons) of the 2,4‐dienoyl‐CoA reductase 1 (*DECR1*) gene, which was induced by a deletion. A schematic diagram of exon extension is shown at the top. The blue segments indicate authentic exons, pink segments indicate extended exons and gray lines indicate introns. The bottom part illustrates the alignment of junction reads around the extended exon in samples with and without the identified indel. The gray segments represent the reads aligned to the genome. The amino acid sequences of the gene before and after the exon extension event are shown below the read alignment. The red box shows the position of the deletion.

In addition to the impact on the creation and use of the canonical splice sites mentioned above, we also found that a case in which a deletion that was able to induce the activation of non‐canonical splice sites. The case was an exon extension event that was considered to be induced by a deletion (from GT to G) and occurred in exon 8 (the 8th of 10 exons) of the 2,4‐dienoyl‐CoA reductase 1 (*DECR1*) gene (Fig. 4). We observed the usage of the non‐canonical GC‐AG splice site in individuals with the deletion; conversely, we did not observe the usage of the same non‐canonical GC‐AG splice site in individuals without this deletion. This extension event may also have introduced a PTC at codon 299 by extending the exon by 4 bp. We considered that the activation of this non‐canonical splice site may be attributed to the GT to G deletion, which can cause the base at the +5‐position relative to this splice site to change from T to G, resulting in the creation of non‐canonical splicing motifs and enhancing splice strength [24]. However, the results of the SpliceAI predictor for this deletion indicated that the possibility of this deletion having an effect on the splice site was very low (the Delta scores for “Donor gain” and “Donor loss” were 0.00 and 0.06, respectively).

Of the remaining 10 cases identified in this study, three were insertions and seven were deletions (Fig. S2). To analyze the potential impact of other SNVs located near the identified novel splice sites, we extracted the SNVs located near (within 10 bp) the 12 identified indels. There was only one SNV located near an indel. This SNV was found to be in linkage with the identified indel, inducing a change from C at position −3 to T at position −2 relative to the donor splice site (Fig. S2E). We considered that this SNV did not create a donor splice site motif, and the use of the novel splice site may be due to the effect of the indel. We found that exonic indels can also induce frameshifts by creating novel splice sites, rather than through variations in the length of the indels, thus leading to the possible PTCs (Fig. S2H).

## Discussion

In this study, we successfully identified 12 exon extension/shrinkage events occurring in 235 normal individuals, together with the associated indels thought to induce these events. Several previous studies have predicted variants affecting the splicing process, including indels, based on deep learning algorithms [20, 25]. In our case, we constructed individual‐specific genomes and transcriptome data and aligned the individual RNA‐seq data to the corresponding individual‐specific genome, to achieve the identification of changes in splice site usage that were induced by indels. The construction of individual‐specific genomes was necessary because of the difficulty in accurately identifying the usage of novel splice sites resulting from indels when mapping RNA‐seq data to the reference genome as it is, as the reference genome cannot reflect the impact of indels on the genomic sequence. To determine the use of novel splice sites, we compared the authentic splice site positions with the mapping positions of the junction reads obtained by mapping. Compared to the identification of SNVs, the impact of the indels on base length results in varying authentic splice site positions across each sample. Therefore, we constructed individual‐specific transcriptome data that reflected the indel information for each individual.

Among the indels identified here, 10 exhibited significantly higher SpliceAI scores (≥ 0.1) compared with those obtained for randomly selected indels, which indicated a higher probability of these indels to affect the usage of splice sites; in contrast, two indels had low SpliceAI scores (< 0.1). For example, we considered that a deletion among the identified indels may induce the generation of a GC splice site motif, leading to the usage of a novel GC splice site, whereas this deletion had a SpliceAI score of 0.00 for “Donor gain” and 0.06 for “Donor loss.” Previous studies have shown that, even though SpliceAI generally performs well in predicting the impact of variants on splice sites compared with other prediction tools, it may still miss a fraction of splice‐affecting variants [26, 27].

Compared with the 371 exon extension/shrinkage events induced by SNVs identified in a previous study [11], we found that the current abnormal splicing events induced by indels accounted for approximately 3.1% of the events. Notably, indels have been confirmed to constitute a certain proportion of pathogenic variants in some diseases. For example, pathogenic variants in the mitofusin 2 (*MFN2*) gene are considered to be associated with Charcot–Marie–Tooth type 2A (CMT2A), with indels contributing to approximately 3.6% of clinical CMT2A cases [28], which is a proportion that is comparable to those identified for exon extension/shrinkage events.

Indels may also induce pseudo‐exon activations by creating novel splice sites within deep intronic regions, leading to the splicing of large coding regions in intronic regions, thus possibly generating abnormal transcripts containing additional exons. In our previous studies about the SNVs‐induced abnormal splicing, we showed that the pseudo‐exon activations occurring at approximately one‐third the rate of exon extension/shrinkage events [10, 11]. Based on this rate, we can estimate that there are likely to be a few cases of pseudo‐exon activation events induced by indels in the same samples. In addition to generating splice site motifs, indels may impact splicing by creating or disrupting splice enhancer and silencer motifs. It is also worth considering the identification and analysis of indels that induce these events above can contribute to the discovery of potential variant functions.

We found that some of the 12 indels identified could impact coding potential. We consider that the potential functional impact of indels that can affect the splicing process, especially those located within intronic regions, might still be overlooked. Moreover, how indels affect the splicing process may determine the extent of their impact on gene function. We consider that when indels create novel splice sites while retaining authentic splice sites, gene function may be preserved even in the presence of homologous indels; however, when indels disrupt authentic splice sites, homozygous occurrences of such indels are likely to affect the gene function, potentially becoming disease‐associated variants. The individuals carrying such indels in heterozygous are not affected but could be carriers of certain diseases. It has been shown that, in contrast to SNVs, the characteristics of indels and the genetic basis for human diseases is still not sufficiently clear [29, 30]. Our findings suggest that the potential functions of some indels have been underexamined. When identifying causative mutations for genetic disorders, it is worth considering indels that may generate or activate the usage of novel splice sites, thereby affecting the usage of authentic splice sites.

## Conflict of interest

The authors declare no conflict of interest.

### Peer review

The peer review history for this article is available at https://www.webofscience.com/api/gateway/wos/peer‐review/10.1002/2211‐5463.13871.

## Author contributions

MS conceived the project. NS conducted material preparation. ZQ performed the main analytical task and wrote the first draft of the manuscript. ZQ, CK and MS performed the interpretation of data. CK and MS reviewed the paper and made critical revisions to the manuscript. All authors contributed to this study and approved the final manuscript.

## Supporting information


**Fig. S1.** Alignment of RNA‐seq data from a sample (NA19248) to the reference genome and the individual‐specific genome.
**Fig. S2.** Examples of identified exon extension/shrinkage events and associated indels.

## Data Availability

The datasets generated during and/or analyzed during the current study are available from the corresponding author on reasonable request. The code developed for this study is freely available at https://github.com/QQZZ2024/Indels‐inducing‐exon‐extension‐and‐shrinkage‐events.
