# Supplementary material for: Identification and analysis of short indels inducing exon extension/shrinkage events
Source: FEBS Open Bio. 2024 Jul 31;14(10):1682–90. doi: 10.1002/2211-5463.13871 (PMC11452298; doi:10.1002/2211-5463.13871)
Supplement: Supplementary file 1 — Fig. S1. Alignment of RNA‐seq data from a sample (NA19248) to the reference genome and the individual‐specific genome. Fig. S2. Examples of identified exon extension/shrinkage events and associated indels. [file FEB4-14-1682-s001.pdf]

Mapping to the reference genome

Mapping to the individual-specific reference genome

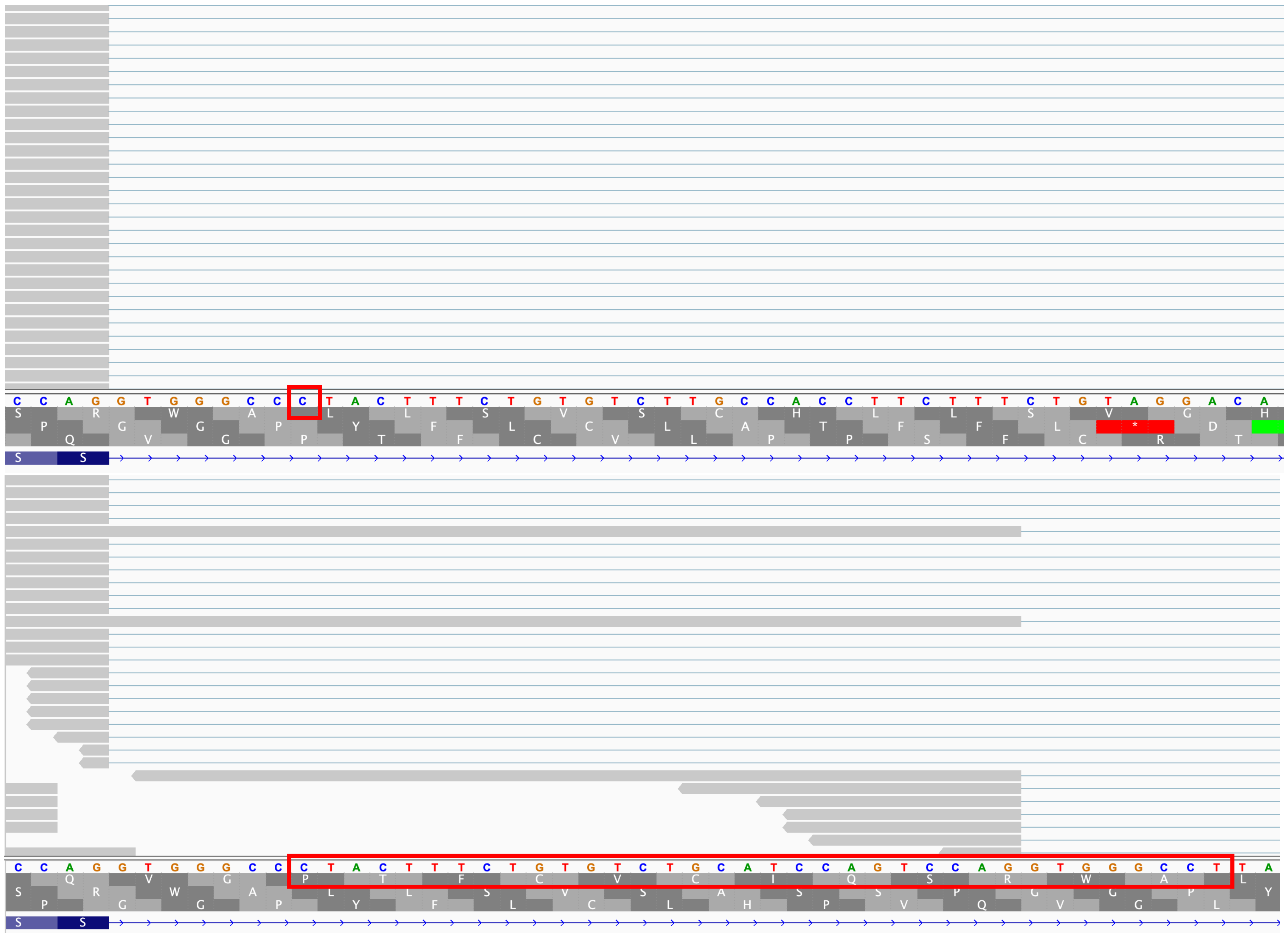

Supplementary Fig. 1. Alignment of RNA-seq data from a sample (NA19248) to the reference genome (top) and the individual-specific genome (bottom). The grey segments represent the reads aligned to the genome. The red box shows the sequences before and after the insertion occurs.

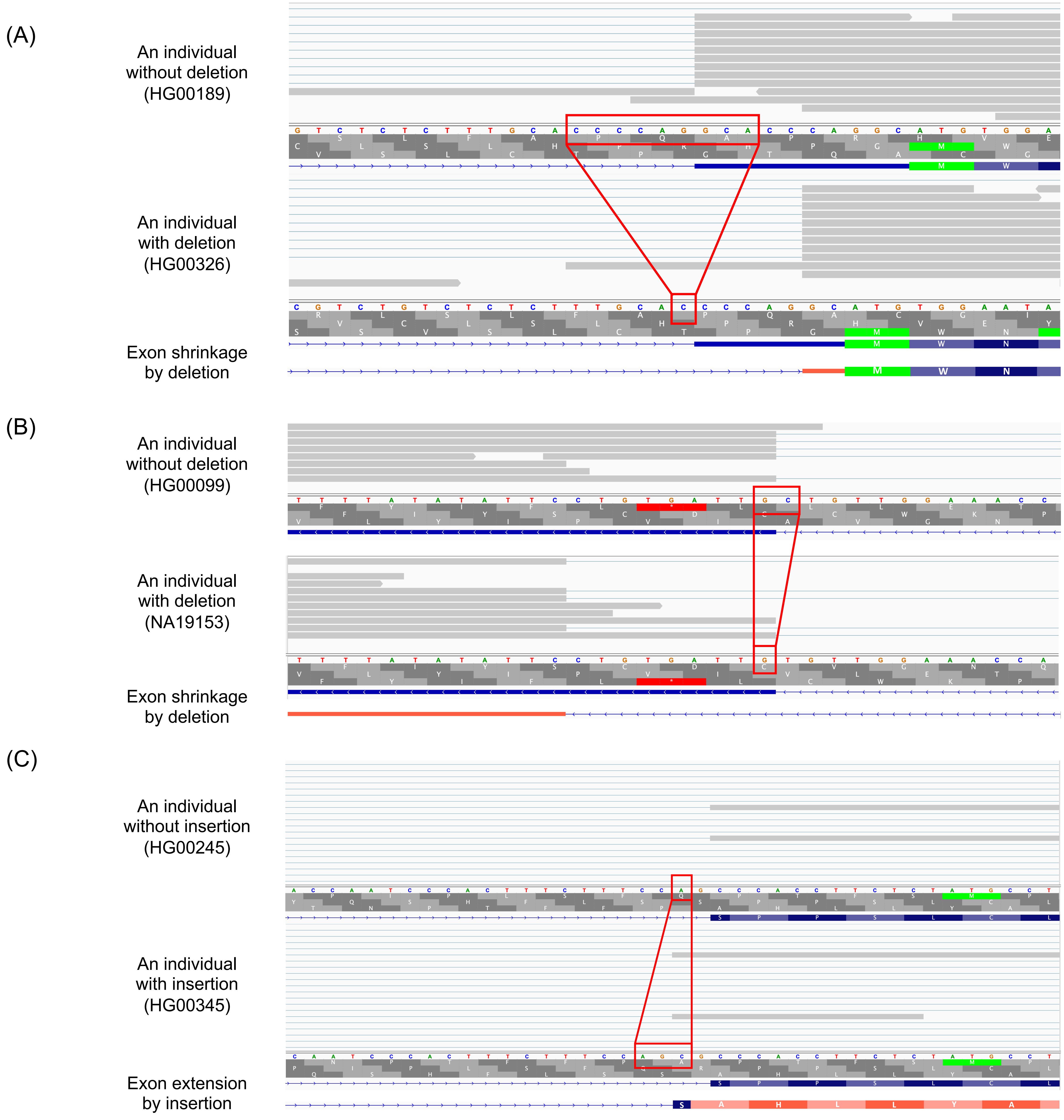

(A)
An individual without deletion (HG00189)
An individual with deletion (HG00326)
Exon shrinkage by deletion
(B)
An individual without deletion (HG00099)
An individual with deletion (NA19153)
Exon shrinkage by deletion
(C)
An individual without insertion (HG00245)
An individual with insertion (HG00345)
Exon extension by insertion

(D)

An individual without deletion (HG00097)

An individual with deletion (NA19108)

Exon shrinkage by deletion

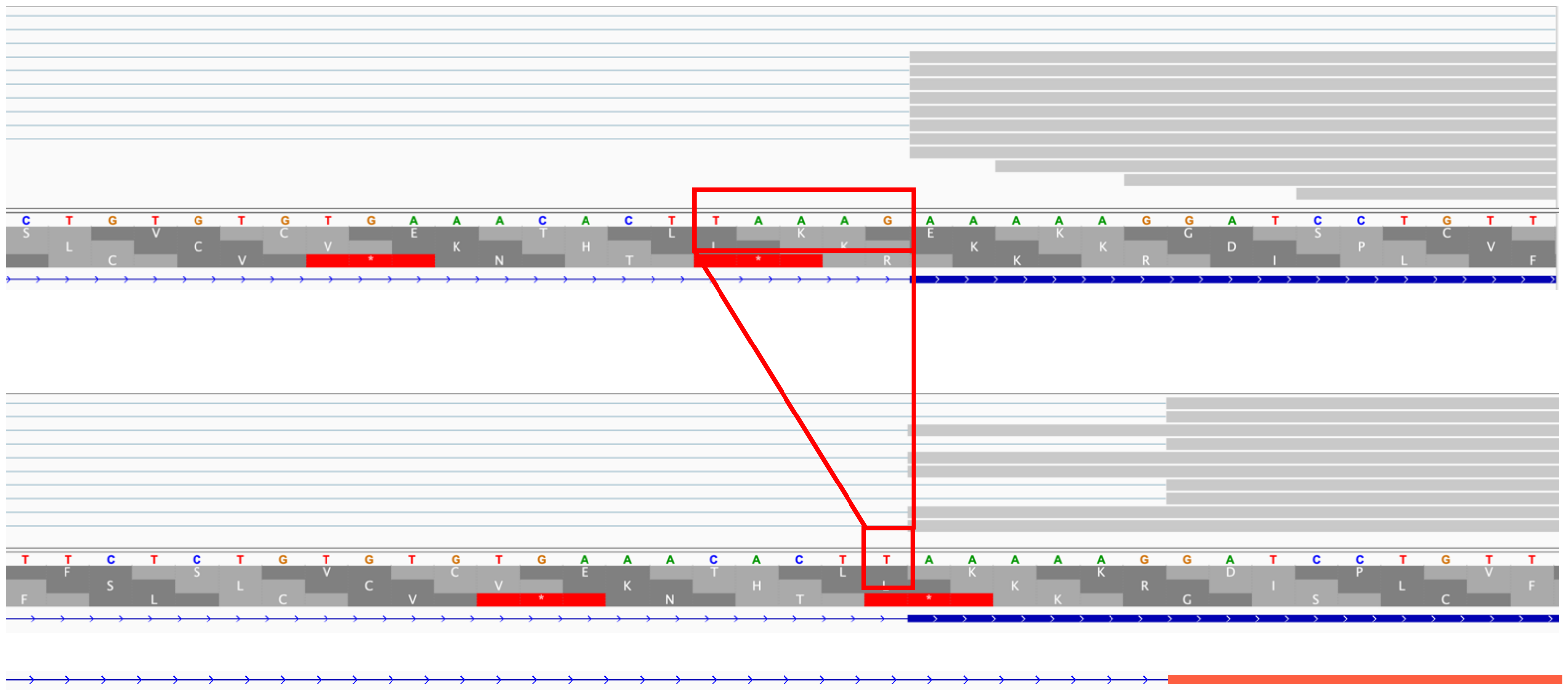

(E)

An individual without deletion (HG00102)

An individual with deletion (NA18861)

Exon shrinkage by deletion

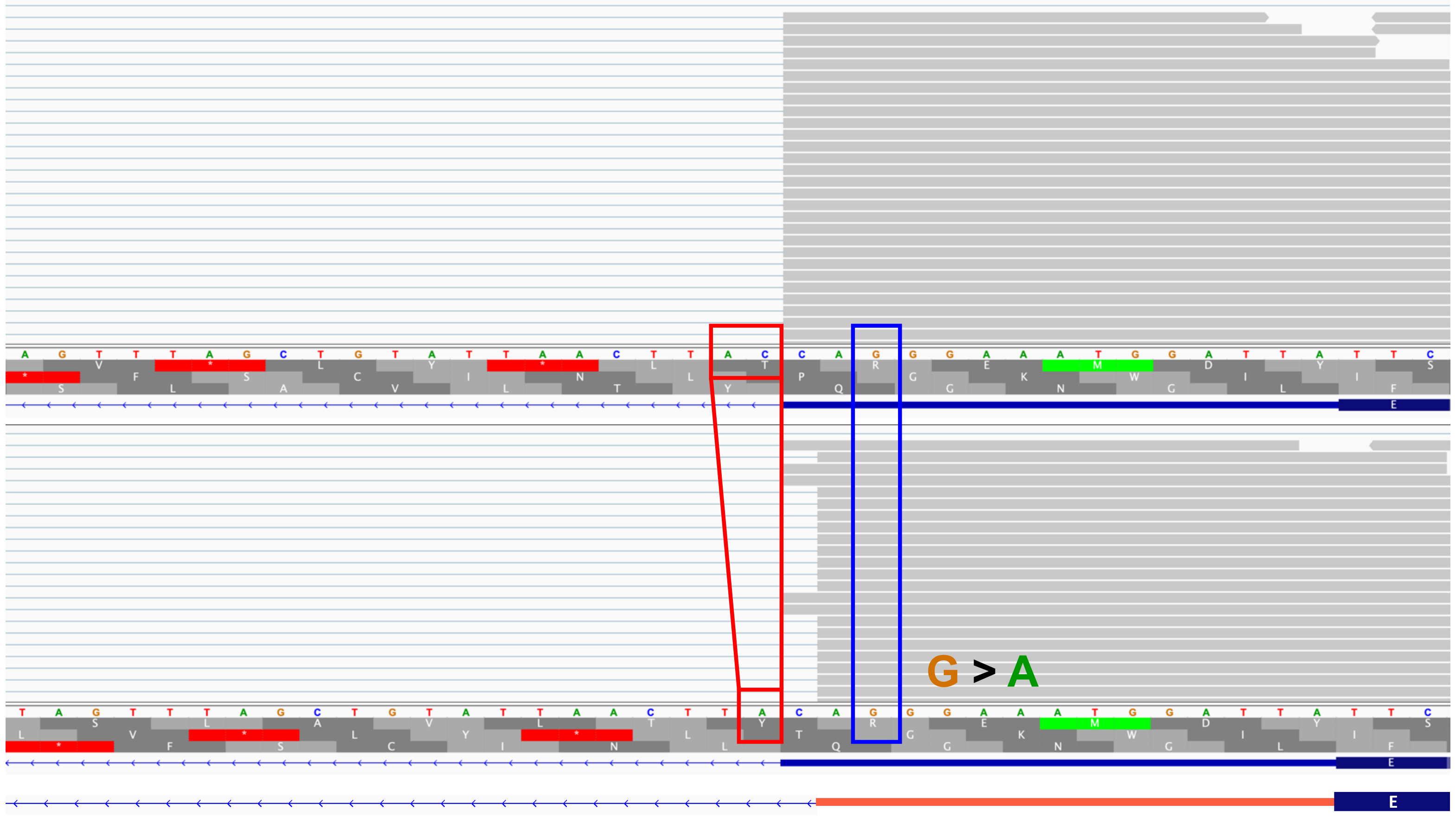

(F)

An individual without deletion (HG00097)

An individual with deletion (HG00111)

Exon shrinkage by deletion

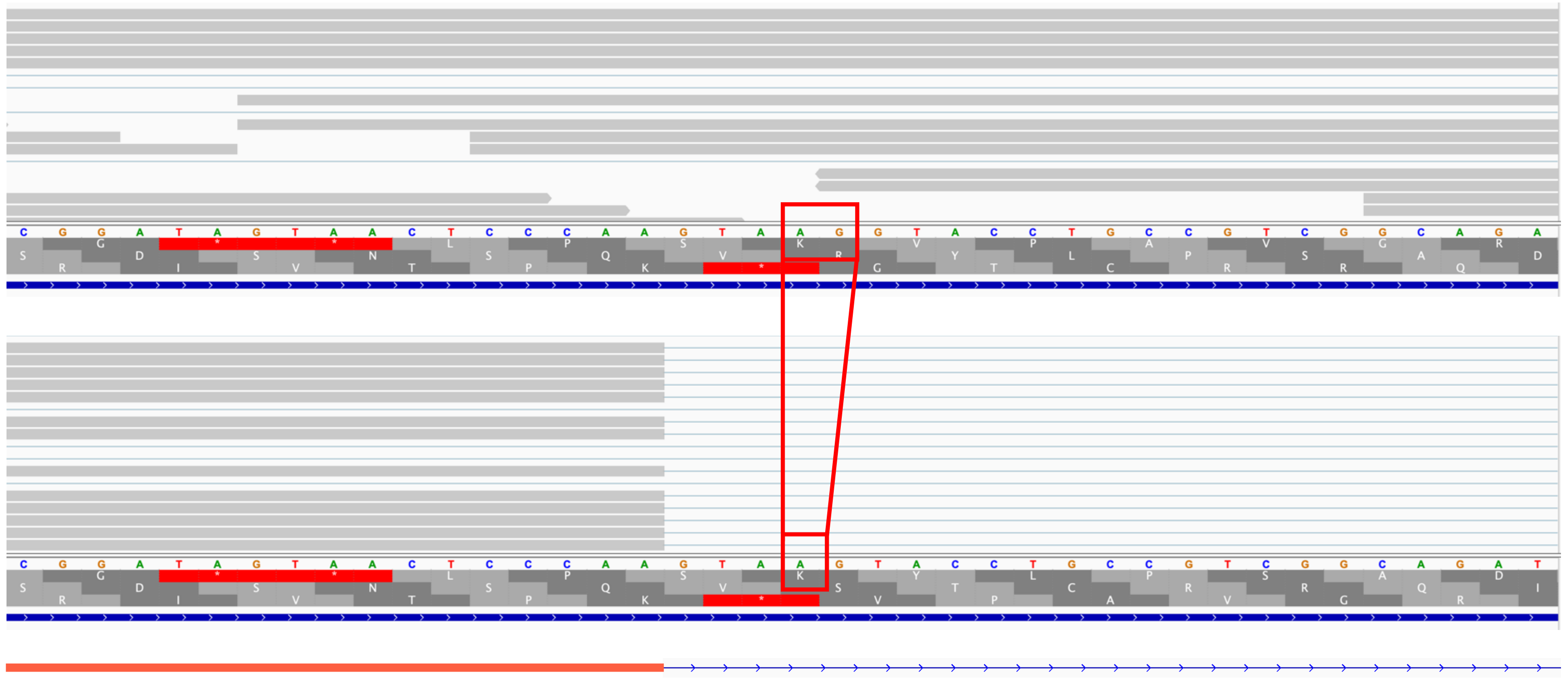

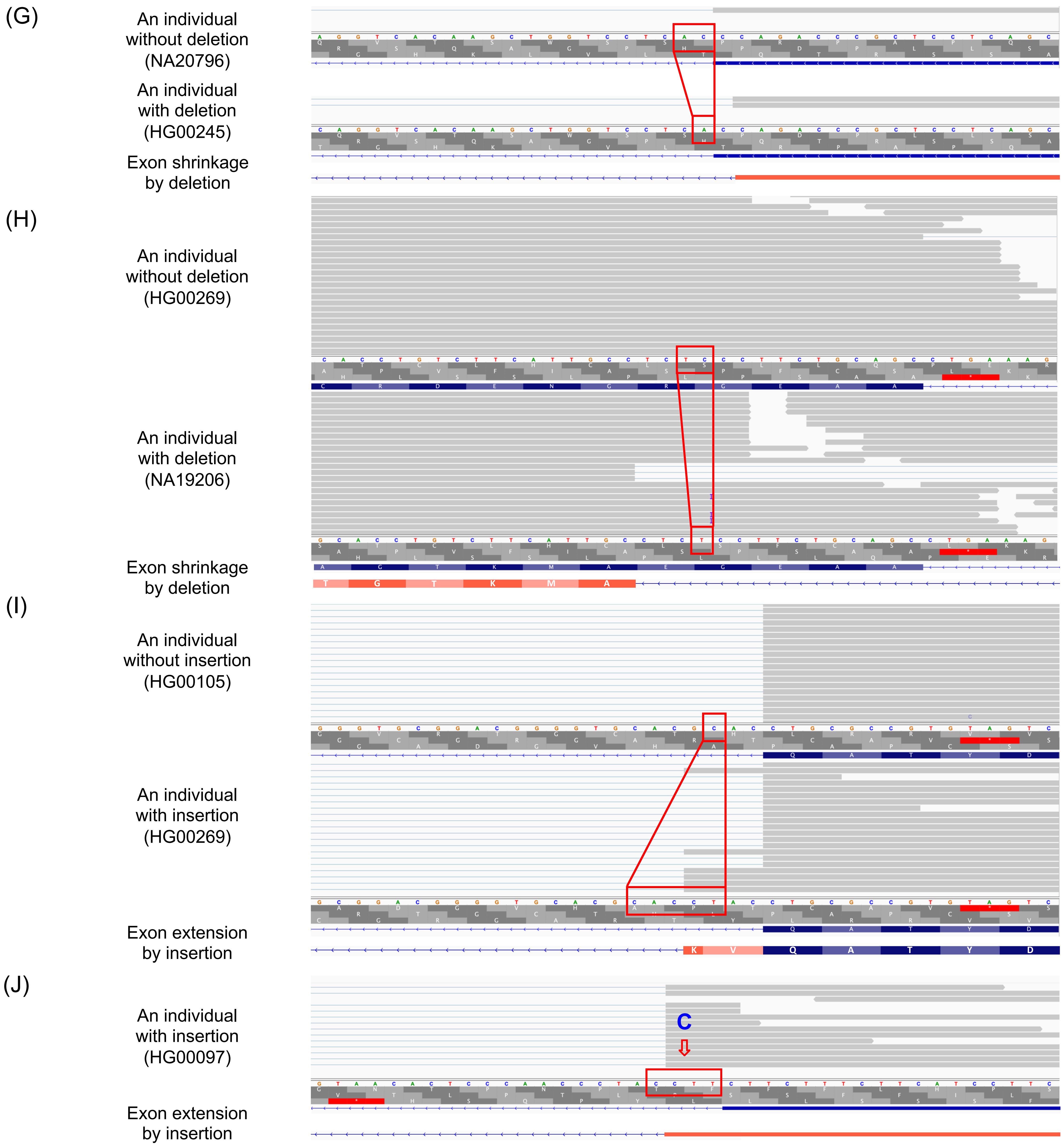

Supplementary Fig. 2. Examples of identified exon extension/shrinkage events and associated indels. Each panel shows the alignment of junction reads around the novel exon boundary in samples with and without the identified indel. The grey segments represent the reads aligned to the genome. The bottom of each panel shows the amino acid sequences of the gene before and after the exon extension/shrinkage event. The red box indicates the position of the associated indel. The blue box indicates the position of SNV near (within 10 bp) the associated indel. Exon extension/shrinkage events occur in *H6PD* (A), *CCNT2-AS1* (B), *CWC27* (C), *POMK* (D), *LPCAT3* (E), *TPT1-AS1* (F), *ENSG00000230805* (G), *PIF1* (H), *MRPS34* (I), *TVP23C-CDRT4* (J), respectively.
